# Allosteric regulation of prostaglandin endoperoxide H_2_ synthases

**DOI:** 10.1016/j.jbc.2025.110927

**Published:** 2025-11-11

**Authors:** Liang Dong, Michael G. Malkowski

**Affiliations:** Department of Structural Biology, Jacobs School of Medicine and Biomedical Sciences, University of Buffalo, the State University of New York, Buffalo, New York, USA

**Keywords:** cyclooxygenase, arachidonic acid, allostery, prostaglandins, ^19^F-NMR

## Abstract

Prostaglandin Endoperoxide H_2_ Synthases (PGHS-1 and PGHS-2), also referred to as cyclooxygenases (COX-1 and COX-2), are homodimeric enzymes that oxygenate arachidonic acid (AA) to generate Prostaglandin H_2_ (PGH_2_), the precursor to prostaglandins, prostacyclin, and thromboxane. The homodimeric enzymes behave as conformational heterodimers comprised of allosteric (E_allo_) and catalytic (E_cat_) subunits. During catalysis, only the E_cat_ subunit actively oxygenates AA to PGH_2_. Different ligands bind to E_allo_ to allosterically modulate the oxygenation of AA in E_cat_. Biochemical studies and functional characterizations have provided compelling evidence for asymmetry between subunits of the homodimer centered at the dimer interface. However, the structural transitions responsible for mediating intersubunit communication remain elusive. This review summarizes the pivotal experiments that have shaped our current understanding of the mechanisms underlying the allosteric modulation of PGHS-1 and PGHS-2. An ensemble-based structural model, derived from one-dimensional fluorine nuclear magnetic resonance spectroscopy, is presented to provide a framework of the conformational landscapes associated with the regulation of PGHS function.

## Overview

Prostaglandins (PG) are bioactive lipids derived from the oxygenation of 20 carbon fatty acids that are integral to the regulation of essential physiological functions, including gastric protection, vascular regulation and blood clotting, renal blood flow and electrolyte balance, inflammation, and reproduction (1). These potent signaling molecules are produced from prostaglandin H_2_ (PGH_2_), which is generated upon the regioselective oxygenation of arachidonic acid (AA; 20:4 ω-6) by the heme-containing, bifunctional enzymes Prostaglandin Endoperoxide H_2_ Synthase-1 and -2 (PGHS-1 and PGHS-2). Abnormal changes in PG production lead to various disease pathologies, including cardiovascular disease, inflammation, and cancer (2). Aspirin, ibuprofen, and other common nonselective nonsteroidal anti-inflammatory drugs (NSAIDs) inhibit the synthesis of PGH_2_ by PGHS-1 and PGHS-2. PGHS-2 is also selectively inhibited by the coxib class of inhibitors that include celecoxib (Celebrex; CBX) and rofecoxib (Vioxx) (3, 4). A brief overview relating the mechanism of catalysis to the structural architecture of homodimeric PGHS is necessary to provide appropriate context into the models developed to describe half-of-sites activity and allosteric regulation of this enzyme. The reader is referred to many excellent reviews that describe in greater detail the enzymology (5, 6, 7, 8), structural biology (9, 10), and pharmacology (11) of PGHS-1 and PGHS-2.

## PGHS structure and function

**Figure 1:**
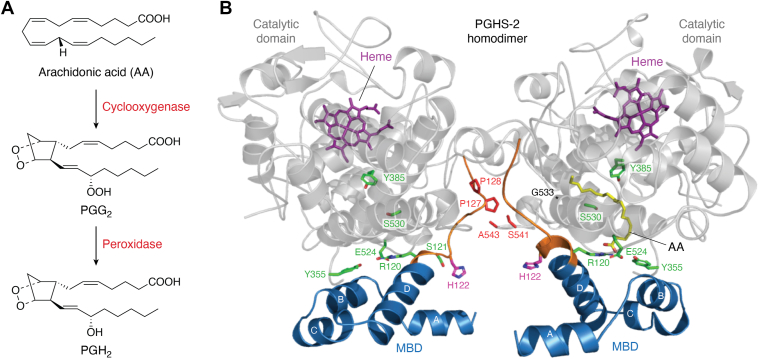
**3D Architecture of the PGHS-2 Homodimer.** (*A*) Catalytic cycle of PGHS depicting the oxygenation of AA to the intermediate PGG_2_*via* the cyclooxygenase activity, followed by reduction of the hydroperoxide group of PGG_2_ by the peroxidase activity to produce PGH_2_. (*B*) Cartoon depiction of the PGHS-2 homodimer structure. The MBD and catalytic domains are colored *blue* and *grey*, respectively. AA (*yellow*) is shown bound within the cyclooxygenase active site of one of the subunits. The side chains of Arg-120, Tyr-355 and Glu-524 are shown as sticks (*green carbons*) at the active site entrance, while the side chain of Tyr-385 and Ser-530 (*green carbons*) are shown at the apex of the active site. The 120 to 129 loop is colored orange, with the side chains of Ser-121 (*green carbons*) and His-122 (*magenta carbons*) shown as sticks. The locations of Pro-127 and Pro-128 (*red carbons*) in one subunit and Ser-541 and Ala-543 (*red carbons*) in the opposite subunit are depicted. Heme (*purple carbons*) is shown bound within the catalytic domain of each subunit. The epidermal growth factor-like domain and small regions of the catalytic domain have been omitted for clarity. Nitrogen and oxygen atoms are colored *blue* and *red*, respectively. Adapted with permission from Dong and Malkowski, Biochemistry 64, 1380 to 1392 (2025). Copyright 2025 American Chemical Society.

PGHS-1 and PGHS-2 convert AA to PGH_2_ *via* sequential cyclooxygenase and peroxidase reactions (Fig. 1*A*). In the first reaction, two molecules of oxygen are incorporated into AA to generate the cyclized intermediate prostaglandin endoperoxide G_2_ (PGG_2_). PGG_2_ is then reduced in the second reaction to form PGH_2_. Initiation of the cyclooxygenase reaction requires a preliminary oxidation of the heme moiety in the peroxidase active site (8). Oxidation of the heme generates an oxy-ferryl porphyrin cation radical that is transferred to Tyr-385 located in the cyclooxygenase active site. The tyrosyl radical abstracts the 13-*proS* hydrogen from AA to initiate multiple rounds of cyclooxygenase catalysis (6).

PGHSs are dimeric enzymes comprised of subunits that are identical in sequence. The subunits form an extensive interaction interface that serves to stabilize the dimeric state of the enzyme (9, 10). Importantly, the PGHS dimer does not dissociate into active subunits. Disruption of the interface utilizing denaturants leads to perturbation of the enzyme fold and the loss of catalytic activity (12). The architecture of each subunit is identical, consisting of three domains: an N-terminal epidermal growth factor-like domain that makes up a significant portion of the dimer interface; a membrane-binding domain (MBD) comprised of four amphipathic α-helices that tether the enzyme to one leaflet of the membrane bilayer; and a globular C-terminal catalytic domain that houses the cyclooxygenase and peroxidase active sites (Fig. 1*B*). The cyclooxygenase active site is located deep within the catalytic domain, while the peroxidase active site, which houses the heme moiety, is situated at its surface. The cyclooxygenase active site is comprised of a long hydrophobic channel that extends upward from the MBD to the base of the heme cofactor, with Tyr-385 located at its apex. A hydrophobic groove is accessible adjacent to Tyr-385, giving the active site channel an overall L-shaped configuration. AA binds within the cyclooxygenase active site with its ω-end buried deep in the hydrophobic groove where it abuts Gly-533 (13). In this conformation, the 13-*proS* hydrogen of AA is optimally aligned below Tyr-385, where it is poised for abstraction to initiate catalysis.

The entrance to the cyclooxygenase active site is demarcated by Arg-120, Tyr-355, and Glu-524, three highly conserved residues that collectively control ligand access and egress (Fig. 1*B*). X-ray crystal structures indicate that when substrate or inhibitor is bound, these residues form an interaction network that decreases the aperture of the entrance to subsequently bury the ligand within the active site (10). Conversely, molecular dynamics simulations have shown that dissociation of this interaction network, predominantly driven by the uncoupling of the ionic interaction between Arg-120 and Glu-524, facilitates the opening of the entrance for release of ligand (14). Arg-120 and Tyr-355 frequently interact with the carboxylate moieties of FAs, as well as carboxylate-containing inhibitors. As such, it has been proposed that the mechanisms underlying allosteric regulation and time-dependent inhibition of PGHSs are correlated with the dynamic movements of these three residues (14, 15, 16, 17, 18, 19).

## Heterodimer technology and the discovery of PGHS-2 half-of-sites activity

As stated above, PGHS enzymes function only as homodimers. Much of the early research surrounding PGHS-1 and PGHS-2 supported the view that each subunit of the homodimer functioned independently. Examination of the structural architecture of both PGHS-1 and PGHS-2 further cemented this view, given that the individual subunits were virtually identical when compared (9, 10). Interestingly, some of the early experimental observations ran contrary to this view. For example, work carried out by Kulmacz and Lands showed that heme binding to a single subunit of PGHS-1 was sufficient to obtain maximal cyclooxygenase activity (20). In follow up experiments, they further showed that indomethacin, flurbiprofen, and meclofenamic acid maximally inhibited PGHS-1 cyclooxygenase activity *via* the binding of the inhibitor to a single subunit (21). These observations suggested that each subunit was “distinct”. Additional pharmacological experiments carried out by Rimon, Rosenstock and colleagues showed that coxibs interfered with the inhibition of PGHS-1 by some NSAIDs without affecting the oxygenation of AA (22, 23, 24). Collectively, these studies were generally ignored due to difficulties rationalizing how the data integrated with the predominating structural and functional models.

Smith and colleagues subsequently set out to determine if each PGHS subunit functions independently or whether crosstalk occurs between subunits of the homodimer. One of the major tools that facilitated this investigation was the utilization of heterodimer constructs (25). As the name implies, constructs of PGHS were engineered such that the dimer was comprised of a “native” subunit, along with a subunit containing a mutation that either abolished the cyclooxygenase activity or attenuated the ability of carboxylic acid-containing NSAIDs to inhibit the enzyme. To generate PGHS heterodimers, two PGHS genes, one of which contained an N-terminal His_6_-tag and one that contained an N-terminal FLAG-tag were inserted behind the polH and p10 promoters, respectively, of the pFastbac Dual vector in the baculovirus expression system. Ensuing expression and post-translational processing in insect cells resulted in the production of three species: His_6_/His_6_ homodimers, FLAG/FLAG homodimers, and His_6_/FLAG heterodimers. Purified heterodimers were then isolated using sequential immobilized metal-affinity and FLAG-affinity chromatographic steps. Subsequent functional characterization confirmed that the His_6_/FLAG heterodimers exhibited virtually identical activity and K_M_ values towards AA when compared to His_6_/His_6_ homodimers (26). (In terms of naming convention, a native PGHS construct with no substitutions in either subunit is defined as Native/Native PGHS, whereas constructs that contain substitutions in both subunits are defined as Mutant/Mutant PGHS. A substitution in a single subunit is defined as Mutant/Native PGHS).

To investigate the interactions between PGHS subunits, two heterodimer constructs were engineered—G533A/Native PGHS-2 and R120Q/Native PGHS-2 (25). The G533A substitution abolishes cyclooxygenase activity through the misalignment of the 13-*proS* hydrogen below Tyr-385 (27, 28). In contrast, the peroxidase activity is not affected indicating that the mutant heterodimer construct is properly folded. If the subunits acted independently, G533A/Native PGHS-2 would be expected to exhibit ∼50% cyclooxygenase activity and 100% peroxidase activity. However, G533A/Native PGHS-2 and Native/Native PGHS-2 exhibited almost the same cyclooxygenase and peroxidase activities, with equivalent K_M_ values for AA. The R120Q mutation retains native-like cyclooxygenase and peroxidase activity but is not inhibited effectively by the time-dependent carboxylic-acid containing inhibitor flurbiprofen (FBP) (29). Again, independently functioning subunits would require FBP to be bound in both cyclooxygenase active sites for complete inhibition of enzyme activity. However, FBP effectively inhibited R120Q/Native PGHS-2 and Native/Native PGHS-2 to similar extents without inhibiting R120Q/R120Q PGHS-2. Collectively, these experiments provided the first evidence that PGHS-2 exhibits half-of-sites activity, with only one of the subunits oxygenating AA at a given time. Given that a structural dimer is required for catalysis, the non-substrate-oxidizing subunit was hypothesized to play an enabling role through changes that occur at the dimer interface (25).

## Fatty acid-mediated crosstalk between PGHS-2 subunits

The establishment of half-of-sites activity confirmed that PGHSs functioned as conformational heterodimers. Subsequent evaluation of AA oxygenation in the presence of different FAs revealed another important caveat of how the conformational heterodimer functions. FAs were found to occupy the cyclooxygenase active site of both subunits at the same time. Moreover, the binding of FA in one subunit altered the cyclooxygenase activity in the partner subunit (30). These studies confirmed that PGHS exhibited crosstalk between subunits, with the subunits of the conformational heterodimer functioning as an allosteric/catalytic couple (E_allo_/E_cat_).

A combination of engineered heterodimers and cross-linking was then utilized to identify putative residues at the dimer interface involved in crosstalk (30). PGHS enzymes contain 12 cysteine residues; 10 are involved in the formation of 5 disulfide bonds, while 2 remain in their free state. A cysteine-free construct (ΔCYS PGHS-2) was engineered and used as a template to build homodimers with inserted cysteine pairs at locations along the dimer interface that were across from one another, where it appeared cross-linking might occur. Disulfide bond formation was then induced by the addition of the oxidant Cu^2+^/*o*-phenanthroline. Of the 5 different cysteine pairs tested, only P126C:A543C ΔCYS PGHS-2 and P127C:S541C ΔCYS PGHS-2 (Fig. 1*B*) led to the formation of cross-linked dimers in the presence of AA and other FA substrates, as well as with common saturated and monounsaturated non-substrate FAs. In contrast, cross-linking was attenuated by some common NSAIDs, including FBP, indomethacin, and ibuprofen.

## Modulation of cyclooxygenase activity by FAs and NSAIDs

Combinations of substrate fatty acids (FAs) and non-substrate FAs were then evaluated to determine how they influenced the oxygenation of AA by PGHS-2 (30, 31). Substrate FAs, including dihomo-γ-linolenic acid (DHLA; 20:3 ω-6), eicosapentaenoic acid (EPA; 20:5 ω-3), linoleic acid (LA; 18:2 ω-6), and α-linolenic acid (alpha-linoleic acid [αLA]; 18:3 ω-3) were found to stimulate the oxygenation of AA. However, the larger stimulatory effects were observed by saturated non-substrate FAs with chain lengths between 12 to 16 carbons. Palmitic acid (PA) exhibited the greatest stimulatory effect, increasing the rate of AA oxygenation 2-fold. For saturated non-substrate FAs with chain lengths greater than 16 carbon atoms, the stimulatory effect decreased with increasing chain length. When the effects of combinations of saturated non-substrate FAs were tested on their ability to stimulate AA oxygenation, the results were additive, suggesting that the non-substrate FAs compete for the same site on PGHS-2 with similar affinities. The addition of non-substrate FAs also did not change the profile of oxygenated products derived from AA (31).

Previous studies had revealed that time-dependent inhibitors such as FBP, CBX, and naproxen (NPX) bind to a single subunit of PGHS to cause maximal inhibition (25, 26). Given that non-substrate FAs also bind preferentially to a single subunit, the interplay between the two different groups of ligands was subsequently investigated. The resulting data indicated that time-dependent inhibitors could be divided into two general classes. For one class, which included CBX, the addition of non-substrate FA either potentiated time-dependent inhibition or had no effect. In contrast, in the other class, which included FBP and NPX, time-dependent inhibition was attenuated.

The experimental findings described above led to the first iteration of the model describing the allosteric modulation of cyclooxygenase catalysis (31). AA and other FA substrates bound to one subunit of PGHS-2 to modulate the catalytic efficiency in the opposite subunit. The modulating subunit was subsequently defined as the allosteric subunit (E_allo_), with the subunit responsible for catalysis defined as the catalytic subunit (E_cat_). Non-substrate FAs do not bind to the E_cat_ subunit, but when they are present in excess, they displace AA from E_allo_. The released AA then becomes available to the E_cat_ subunit for oxygenation. The crystal structure of PA bound to PGHS-2 also provided support for the model, as the cyclooxygenase active site of only one of the two subunits was occupied with PA (31). Time-dependent inhibitors also bound to a single subunit to elicit their effects. The profiles described above that differentiate the inhibitors into the two general classes also indicated to which of the subunits they bound. FBP and NPX bound to the E_allo_ subunit, given that non-substrate FAs attenuate their inhibitory responses, whereas CBX bound to E_cat_, given that non-substrate FAs do not alter their inhibitory actions (31).

## Pre-existent asymmetry in the PGHS-2 sequence homodimer

Based on the initial observations derived from examination of the G533A/Native PGHS-2 and R120Q/Native PGHS-2 constructs, as well as the modulation of cyclooxygenase activity by non-substrate FAs, Smith and colleagues hypothesized that the binding of a ligand was responsible for locking each subunit into its catalytically competent state. They proposed that each monomer of the dimer fluxed between two forms, E_allo_/E_cat_ and E_cat_/E_allo_. The binding of heme and/or ligand would slow or prevent the flux and stabilize the dimer into its E_allo_/E_cat_ state (26). To test this hypothesis, Smith and colleagues examined the heme binding affinity, cyclooxygenase activity, and inhibition profiles of additional heterodimer constructs (26, 31).

UV spectroscopy coupled with the evaluation of cyclooxygenase activity of native and heterodimer PGHS-2 constructs revealed that heme bound with high affinity to one subunit and with lower affinity to the other subunit. Heterodimer constructs containing R120A, Y385F, or S530A substitutions in a single subunit exhibited roughly equivalent affinities for heme when compared to the native subunit. When the cyclooxygenase activity was evaluated in the presence of different concentrations of heme, maximal activity was observed at a ratio of ∼1 heme per dimer, consistent with the early work carried out on PGHS-1. Heme binding was also shown to be unaffected by substrate and non-substrate FAs and NSAIDs. The observation of a high affinity heme binding site led to defining the subunit containing the high affinity heme site as the catalytic subunit (E_cat_) and partner subunit as the allosteric subunit (E_allo_).

One of the pivotal heterodimer constructs examined to further test the hypothesis was S530A/Native PGHS-2. Ser-530 lies at the apex of the cyclooxygenase channel below Tyr-385 and is the target of acetylation by aspirin. Aspirin is the only NSAID that covalently binds to PGHS, rendering PGHS-1 completely inactive, while changing the product profile of PGHS-2 from a cyclooxygenase to a lipoxygenase (32). The loss of the hydroxyl group in the S530A mutation does not allow acetylation by aspirin. Evaluation of aspirin-labelling of S530A/Native PGHS-2 in the absence of heme surprisingly showed that only half of the native subunits were acetylated, when it was expected that all native subunits would be acetylated. This suggested that in solution in the absence of heme that the S530A/Native heterodimer existed as a stable mixture of E_allo_-S530A/E_cat_-Native and E_cat_-S530A/E_allo_-Native (26).

Additional heterodimer constructs, including Y385F/Native PGHS-2, R120A/Native PGHS-2, and R120Q/Native PGHS-2, exhibited patterns of heme affinity, inhibitor sensitivity, and cyclooxygenase activity that were consistent with a pre-existent assignment of E_allo_ and E_cat_ rather than the originally proposed flux model. As a result, the initial model describing the allosteric modulation of cyclooxygenase catalysis was modified to include this new finding. In the updated model, PGHS-2 is initially translated into mixtures of dimers. Subsequent folding and processing, including the binding of heme, define the E_cat_ subunit, which subsequently locks the dimer into a pre-existent conformational heterodimer. Once a conformational heterodimer is formed, the subunits do not flux between E_allo_ and E_cat_.

There are important caveats associated with this model. First, it is unknown what structural changes occur upon heme binding. As discussed below, crystal structures of PGHS have been determined utilizing an enzyme that has been reconstituted with heme concentrations that are well above the K_d_ values of both the high-affinity and low-affinity binding sites on the subunits. Subsequent analyses do not reveal differences between subunits of the dimer. Second, heme may act as a co-chaperone during folding and processing, such that its binding to one subunit influences the distribution of E_allo_ *versus* E_cat_ in the resulting conformational heterodimer. Indeed, a correlation of relative heme binding affinity *versus* the proportion of subunits in the E_cat_ form is observed when a subunit containing an R120, Y385, or S530 substitution is paired with a native subunit.

## Defining E_allo_ and E_cat_ using a Y385F/Native PGHS-2 heterodimer

One of the most useful constructs designed by Smith and coworkers to evaluate allosteric regulation of PGHS by substrate and non-substrate FAs, NSAIDs, and coxibs was the Y385F/Native PGHS-2 heterodimer (26). As noted earlier, Tyr-385 is responsible for abstracting the 13-*pro*S hydrogen from AA to initiate cyclooxygenase catalysis. While Y385F/Y385F PGHS-2 lacks cyclooxygenase activity, Y385F/Native PGHS-2 exhibited V_max_ values that were >90% that of native/Native PGHS-2, with equivalent K_m_ values when AA was utilized as the substrate. Maximal activity was also observed for Y385F/Native PGHS-2 with one high-affinity heme bound per dimer, presumably to the E_cat_-Native subunit and consistent with that observed for Native/Native PGHS-2. However, the K_d_ value for this high-affinity heme binding site was 0.35 μM, slightly lower than the reported value of 0.15 μM for the native construct. The Y385F/Native heterodimer oxygenated the alternative FA substrates EPA, DHLA, and LA at similar levels to that observed for Native/Native PGHS-2. Non-substrate FAs also stimulated the cyclooxygenase activity of Y385F/Native PGHS-2 in an analogous manner to that observed for Native/Native PGHS-2, with PA serving as the most efficient activator. The potencies of time-dependent inhibition by FBP, NPX and CBX were similar for both the Y385F/Native and Native/Native PGHS-2 constructs. PA attenuated time-dependent inhibition of the Y385F/Native PGHS-2 construct by FBP and NPX, consistent with the inhibitors binding solely to E_allo_ to inhibit cyclooxygenase activity. In contrast, PA potentiated time-dependent inhibition by CBX for both constructs, consistent with CBX binding solely to E_cat_. Collectively, these results indicate that the subunits comprising the Y385F/Native heterodimer construct could be clearly defined, with the Y385F-containing subunit functioning as E_allo_ and the native subunit functioning as E_cat_ (E_allo_-Y385F/E_cat_-Native). Moreover, the heme-binding, kinetic, allosteric potentiation, and inhibition profiles are similar to that observed for Native/Native PGHS-2.

## A S121P mutation within the 120 to 129 loop alters its position and allosteric regulation of PGHS-2

The initial Cu^2+^/*o*-phenanthroline crosslinking studies suggested that ligand binding to E_allo_ altered the position of a 10-residue region at the dimer interface lying just downstream of the cyclooxygenase active site entrance. The Native/Native homodimer and E_allo_-Y385F/E_cat_-Native heterodimer constructs were utilized to make mutations to residues within this region, hereafter referred to as the 120 to 129 loop (Fig. 1*B*). Subsequent characterization of AA oxygenation in E_cat_ in the presence of allosteric potentiators and inhibitors provided a readout of the allosteric effects of ligand binding to E_allo_ and by extension the role that each residue played in the modulation of catalysis. A serine to proline mutation at position-121 (S121P/S121P PGHS-2) was identified that exhibited twice the V_max_ of the native enzyme (17). Moreover, this mutant was insensitive to potentiation by PA and inhibition by FBP and NPX. Ser-121 is located in the fourth helix of the MBD, where it is positioned adjacent to Arg-120 at the cyclooxygenase active site entrance (Fig. 1*B*). Determination of the unliganded S121P/S121P PGHS-2 crystal structure revealed that the mutation caused a 3 Å displacement of the main chain and unwinding of the local helical secondary structure, resulting in the disordering of the side chain of Arg-120 (17). Unfortunately, crystal lattice contacts precluded the ability to identify additional structural differences within the 120 to 129 loop. Interestingly, when the S121P mutation was introduced into the P127C:S541C ΔCYS PGHS-2 cross-linking construct, spontaneous cross-linking occurred during protein production and in the absence of oxidant. Given that spontaneous cross-linking could not be prevented by adding FBP or other inhibitors during protein expression, it was hypothesized that the S121P substitution caused a repositioning of the 120 to 129 loop at the dimer interface (31).

Constructs that had the S121P mutation in either E_allo_ or E_cat_ were then functionally characterized to further evaluate how this mutation enabled the observed increase in V_max_. Locating the S121P substitution in E_allo_ was shown to be primarily responsible for the observed 2-fold potentiation in cyclooxygenase activity, with a smaller but measurable effect observed when the substitution was located in E_cat_. The E_allo_-S121P:Y385F/E_cat_-Native heterodimer was less sensitive to stimulation by PA compared to the E_allo_-Y385F/E_cat_-Native construct. In contrast, the relative increase in activity observed for PA stimulation of the E_allo_-Y385F/E_cat_-S121P heterodimer construct was identical to that seen for the E_allo_-Y385F/E_cat_-Native construct. Evaluation of time-dependent inhibition utilizing FBP showed a similar pattern. The magnitude of inhibition of E_allo_-S121P:Y385F/E_cat_-Native was attenuated compared to E_allo_-Y385F/E_cat_-Native and E_allo_-Y385F/E_cat_-S121P. Conversely, inhibition of E_allo_-S121P:Y385F/E_cat_-Native utilizing an inhibitor that functions by competing solely for E_cat_ was unaffected. The S121P heterodimer constructs were also engineered to place an R120A mutation in E_allo_ and subsequently evaluated for their ability to potentiate activity in the presence of PA and inhibit activity in the presence of FBP. Heterodimer constructs containing the R120A mutation are unable to bind PA and FBP and thus are unable to respond to these allosteric effectors. When the R120A mutation was introduced into this background (E_allo_-R120A:S121P:Y385F/E_cat_-Native), the construct was not inhibited by FBP, while the presence of S121P in E_allo_ increased the V_max_ by more than threefold. These outcomes were consistent with the hypothesis that the S121P substitution in E_allo_ acted by supplanting the allosteric responses to AA, PA, and allosteric inhibitors (17).

The allosteric effects observed for the S121P substitution in E_allo_ provided an additional key piece to the model for the allosteric regulation of PGHS—the involvement of the 120 to 129 loop in mediating the allosteric responses to FAs and inhibitors. Moreover, it identified that ligand binding and/or mutations to E_allo_ could modulate the V_max_ for PGHS-2 activity over at least a 20-fold range (17). Presumably, the degree and manner in which the 120 to 129 loop interacts with E_cat_ are what modulates cyclooxygenase activity in E_cat_.

## Allosteric regulation of PGHS-1

Only a handful of studies have evaluated allosteric regulation of AA oxygenation in PGHS-1. PGHS-1 and PGHS-2 display ∼60% sequence identity, catalyze the same reactions, and exhibit virtually identical structural architecture (6, 9, 10). Like PGHS-2, PGHS-1 behaves as a conformational heterodimer in solution, exhibiting half-of-sites activity through an allosteric/catalytic coupling between subunits (30, 33). Heme binds with high-affinity to one subunit and low affinity to the partner subunit of PGHS-1, with maximal cyclooxygenase activity observed at a heme ratio of ∼1 per dimer. In contrast to PGHS-2, non-substrate FAs inhibit AA oxygenation by PGHS-1 with little specificity (34). Maximal inhibition of 55% is observed in the presence of PA, stearic acid (18:0), and oleic acid (18:1 ω-9). Inhibition occurs *via* non-substrate FA binding to E_allo_ rather than competing with AA for binding to E_cat_. While PGHS-2 exhibits a promiscuous substrate specificity, AA and DHLA are the preferred substrates for PGHS-1 (6). EPA, DHA, and other ω-3 and ω-6 FAs are modest inhibitors of PGHS-1. Non-substrate FAs somewhat augment the inhibitory effects of EPA and DHA on the oxygenation of AA by PGHS-1. It has been postulated that non-substrate FAs are important in the coordinate regulation of PGHS-1 *versus* PGHS-2 when the two isoforms are coexpressed at comparable levels in cells (34, 35). Non-substrate FAs have been shown to modestly potentiate time-dependent inhibition of PGHS-1 by FBP, NPX, aspirin, and diclofenac, indicating that the inhibitors act by binding to the E_cat_ subunit of PGHS-1.

While the tertiary structures of PGHS-1 and PGHS-2 are virtually superimposable, there are subtle but significant differences in the primary sequences of PGHS-1 *versus* PGHS-2. There is only 39% sequence identity between the MBDs, with PGHS-2 containing a proline insertion between the C and D helices that is absent in PGHS-1. Within the center of the 120 to 129 loop, residue 125 is a proline in PGHS-1 and an aspartate in PGHS-2. Finally, the substitutions of cyclooxygenase active site residues Ile-434, His- 513, and Ile-523 in PGHS-1 to Val-434, Arg-513, and Val-523 in PGHS-2 generate a 25% increase in the volume of the cyclooxygenase active site of PGHS-2, resulting in the formation of a side pocket that has been exploited in the design of coxibs (9). Some combination of these differences may contribute to differential allosteric regulation observed between isoforms.

## Correlating structure with function using solution-based ^19^F-NMR

The establishment of PGHS behaving as a conformational heterodimer functioning cooperatively has prompted a search to identify the transient structural states that are central to the mechanisms associated with allosteric modulation of activity. Traditionally, X-ray crystal structures of native and mutant forms of PGHS-1 and PGHS-2 bound with substrates and inhibitors have provided significant insight into structure-function relationships governing many of the fundamental features associated with PGHS catalysis and inhibition (9, 10). Most of these structures have been solved in orthorhombic space groups that contain a homodimer in the asymmetric unit (9). As such, a major limitation that has routinely been encountered in analyzing these structures has been the striking similarity observed across complexes, regardless of the presence or type of ligand bound (10). This uniformity likely arises during crystal formation, where crystal lattice contacts restrict conformational sampling and instead stabilize a single ensemble that may be only sparsely populated in solution. Consequently, these static snapshots have offered limited insight into the conformational motions governing allosteric modulation.

To address this challenge, ^19^F-nuclear magnetic resonance spectroscopy (^19^F-NMR) was employed to probe PGHS-2 in solution in the presence of different ligands, where it is free to undergo the conformational motions that provide authentic regulation of the catalytic activity. ^19^F-NMR is highly sensitive to structural and environmental changes and can identify structural changes in targeted regions of a protein that are undetected using conventional NMR or X-ray crystallographic methods. This technique provides advantages over traditional solution NMR given that ^19^F is not naturally incorporated into proteins, the ^19^F nucleus exhibits similar sensitivity for NMR detection to that of protein nuclei, and ^19^F labeling results in one-dimensional spectra that are greatly simplified by a reduction in spatial crowding (36, 37).

An H122C mutation was engineered into the ΔCYS PGHS-2 homodimer construct (H122C/H122C ΔCYS PGHS-2) to facilitate labeling of the enzyme with the thiol-reactive compound 3-bromo-1,1,1-trifluoroacetone (BTFA) (18). The decision to position the label at position-122 stems from the fact that His-122 lies adjacent to Arg-120 at the cyclooxygenase channel entrance, is part of the 120 to 129 loop, and is fully solvent exposed (Fig. 1*B*). Consequently, ligand-induced movements would be expected to alter the local environment of the TFA label, leading to changes in the observed states in the presence and absence of ligands.

## Tightened and relaxed states of the cyclooxygenase active site entrance are linked to interactions with and between Arg-120, Tyr-355, and Glu-524

**Figure 2:**
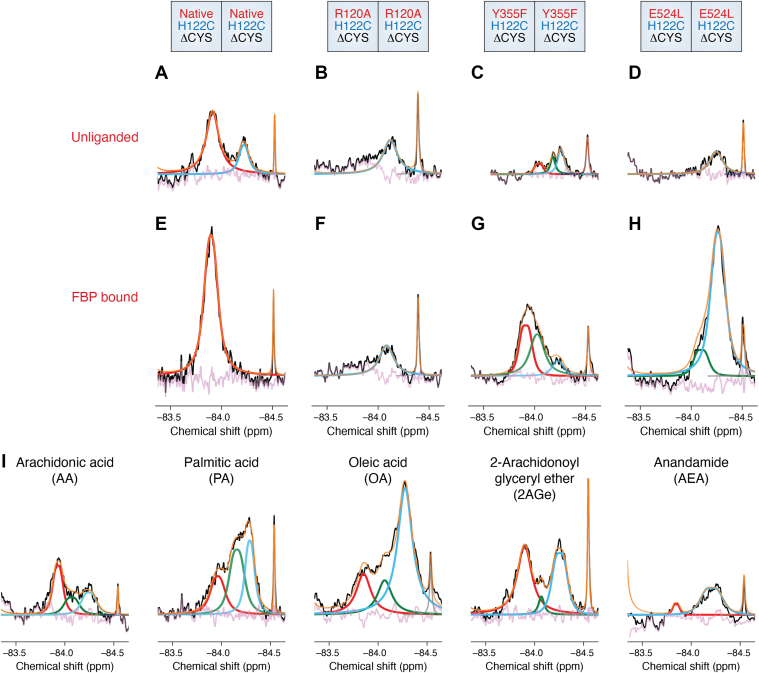
**^19^F-NMR Evaluation of Native and Mutant Homodimer Constructs in the Presence of FBP, AA, and Allosteric Modulators.** Comparison of ensembles arising from native and mutant constructs in unliganded (*A*–*D*) and FBP-bound (*E*–*H*) states. The constructs utilized in the ^19^F-NMR experiments are depicted at the *top* of the figure. *I*, ensembles arising from H122C/H122C ΔCYS PGHS-2 in the presence of arachidonic acid (AA), palmitic acid (PA), oleic acid (OA), 2-arachidonoyl glyceryl ether (2AGe), and anandamide (AEA). Experimental spectra were deconvoluted and fit to a general Lorentzian function as described in (18). The fitted peaks are colored to represent the ensembles, with S1, S2, and S2a colored *red*, *cyan*, and *green*, respectively. Adapted with permission from Dong and Malkowski, Biochemistry 62, 3134 to 3144 (2023). Copyright 2023 American Chemical Society.

^19^F-NMR analysis of unliganded H122C/H122C ΔCYS PGHS-2 resulted in the observation of two major ensembles, hereafter referred to as S1 and S2, respectively (Fig. 2*A*) (18). These ensembles were postulated to represent tightened (S1) and relaxed (S2) states of the cyclooxygenase active site entrance based on previously published structural and molecular dynamics studies, along with the location of the label adjacent to Arg-120. The tightened state of the entrance is generated by the formation of the interaction network between Arg-120, Tyr-355, and Glu-524. This state is observed in virtually all crystal structures of PGHS solved to date and thus is referred to as the crystallographic pose. Conversely, the relaxed state is induced upon the uncoupling of the interaction between Arg-120 and Glu-524. The binding of ligands that interact with Arg-120, such as FBP, caused a complete shift to the tightened state at the active site entrance (Fig. 2*E*). Indeed, when an R120A mutation was engineered into the H122C/H122C ΔCYS PGHS-2 construct, the S1 state was not observed in the unliganded or FBP bound states, further confirming the destabilization of the tightened state (Fig. 2, *B* and *F*). Evaluation of the E524L mutation in the unliganded and FBP bound states resulted in a predominant S2 state, which was expected due breakage of the ionic interaction between this side chain and Arg-120 and as a consequence, the inability to form the tightened state (Fig. 2, *D* and *H*). The S1 and S2 states were also observed when the Y355F mutation was evaluated in its unliganded and FBP bound states, along with a third state, hereafter referred to as S2a (Fig. 2, *C* and *G*). The Y355F mutation in PGHS-2 has been postulated to alter allosteric regulation, through shifts in the equilibrium between the tightened and relaxed states of the cyclooxygenase channel entrance (15). Thus, it was hypothesized that the S2a state was related to conformational motion(s) associated with the modulation of enzyme activity (18).

## AA and allosteric ligands enhance S2a

The productive conformation of AA binds within the cyclooxygenase active site of PGHS-2 with its carboxylate group located near its entrance (Fig. 1*B*) (38). However, the carboxylate group does not form stabilizing interactions with Arg-120. Instead, Arg-120 forms an ionic interaction with Glu-524 to enclose the substrate within the active site. ^19^F-NMR analysis of H122C/H122C ΔCYS PGHS-2 bound with AA resulted in the observation of tightened and relaxed states, as well as S2a (Fig. 2*I*). Given that AA is an allosteric potentiator of the cyclooxygenase activity of PGHS-2, the observation of S2a is in line with this state being involved in the allosteric modulation of the enzyme. To further probe the origin of S2a, spectra derived in the presence of allosteric and non-allosteric ligands were evaluated. The allosteric ligands PA and OA potentiated the S2a signal, whereas the non-allosteric substrate 2-arachidonoyl glyceryl ether weakly potentiated S2a, while anandamide did not, providing additional support for this assignment (Fig. 2*I*).

## Conformational asymmetry between subunits and its role in the allosteric regulation of PGHS-2

The evaluation of ^19^F-NMR spectra derived from the H122C/H122C ΔCYS PGHS-2 homodimer construct, in conjunction with the use of site-directed mutants and ligands producing known functional outcomes, demonstrated the ability to link these outcomes to specific conformational landscapes. However, as the free cysteine was engineered into both subunits of the PGHS-2 homodimer, the resulting conformational states represented a composite of states derived from both E_allo_ and E_cat_. Heterodimer constructs that placed the free cysteine in E_allo_ or E_cat_ were subsequently engineered to gain a clearer understanding of the states arising in each subunit in the presence of allosteric potentiators and inhibitors (19).

**Figure 3:**
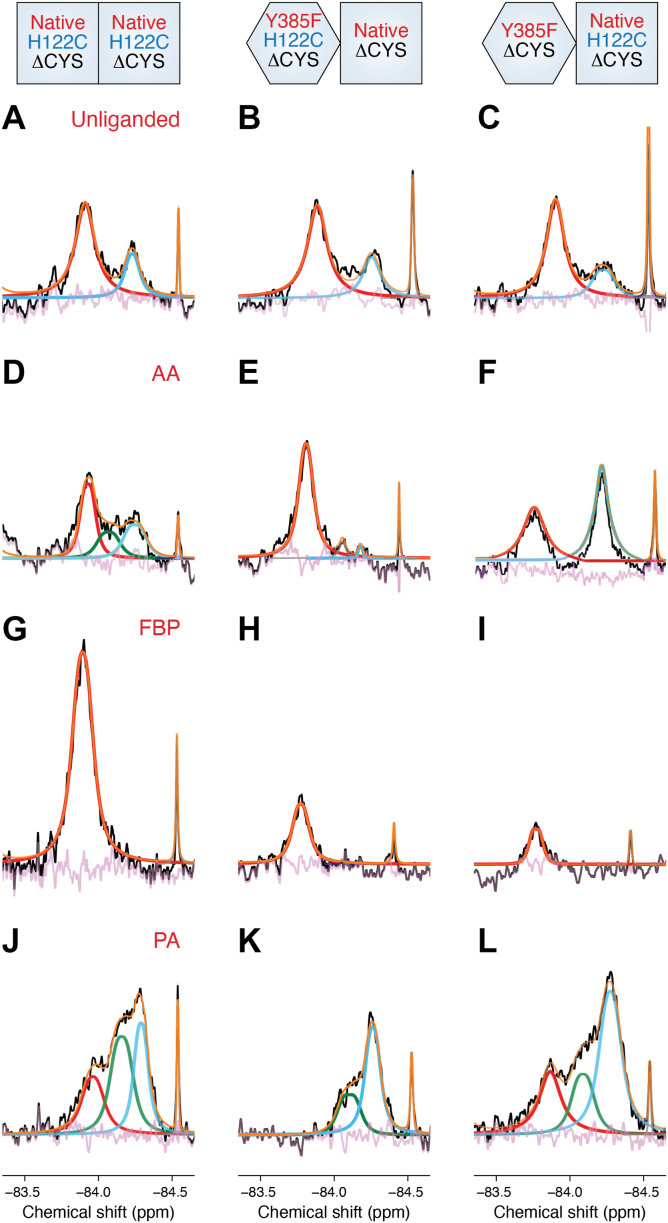
**^19^F-NMR Spectra Derived from Heterodimer Constructs bound with AA, FBP, and PA.** Comparison of ensembles arising from the native homodimer and heterodimer constructs in unliganded (*A*–*C*), AA-bound (*D*–*F*), FBP-bound (*G*–*I*), and PA-bound (*J*–*L*) states. Experimental spectra colored, deconvoluted and fit as described in Figure 2. The constructs utilized in the ^19^F-NMR experiments are depicted at the top of the figure. Adapted with permission from Dong and Malkowski, Biochemistry 64, 1380 to 1392 (2025). Copyright 2025 American Chemical Society.

^19^F-NMR analyses of the heterodimer constructs revealed distinct conformational states at the cyclooxygenase active site entrance depending on the ligand bound to E_allo_. As expected, we observe both S1 and S2 states in the unliganded heterodimer constructs, identical to that seen in H122C/H122C ΔCYS PGHS-2 (Fig. 3, *A*–*C*). In contrast to the homodimer construct, AA binding induced marked asymmetry between subunits, with E_allo_ adopting a tightened state and E_cat_ predominantly adopting a relaxed state (Fig. 3, *D*–*F*). The binding of PA and FBP to the heterodimer constructs, which modulate AA oxygenation *via* binding to E_allo_, resulted in the loss of asymmetry at the active site entrance between E_allo_ and E_cat_. FBP binding stabilized the tightened state in both subunits (Fig. 3, *G*–*I*). The interaction with Arg-120 was also required in E_allo_, as the R120A mutation prevented allosteric inhibitor-induced formation of the tightened state and attenuated time-dependent inhibition (19). The observation of tightened states in both E_allo_ and E_cat_ indicates that inhibitor binding impedes the structural transitions that drive intersubunit communication, facilitating catalysis. In contrast, PA binding resulted in the stabilization of relaxed states in both subunits (Fig. 3, *J*–*L*). The S2a state was also observed, further suggesting that there are additional structural changes invoked at the dimer interface that facilitate the stabilization of the relaxed state in E_cat_.

## Subunit-specific placement of S121P alters the conformational landscape to facilitate potentiation of PGHS-2 activity

**Figure 4:**
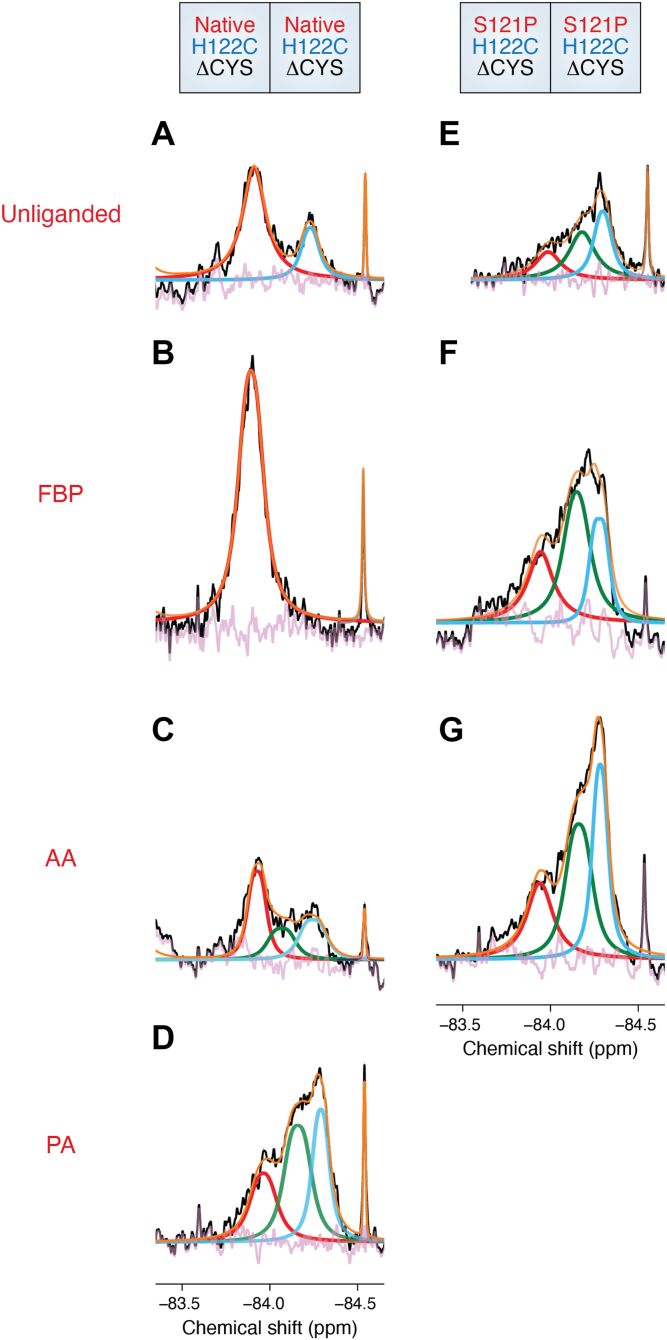
**^19^F-NMR Spectra Derived from Native and S121P Homodimer Constructs.** Comparison of ensembles arising from native and S121P homodimer constructs in unliganded (*A*, *E*), FBP-bound (*B*, *F*), and AA-bound (*C*, *G*) states. *D*, PA bound to the native homodimer construct. Experimental spectra colored, deconvoluted and fit as described in Figure 2.

^19^F-NMR analysis of the S121P/S121P ΔCYS PGHS-2 construct was carried out to evaluate how this mutation affected the distributions of the S2a state (19). As discussed above, the S121P mutation yields a variant that has twice the V_max_ of native PGHS-2 and is insensitive to allosteric potentiation by PA and allosteric inhibition by FBP. In contrast to the unliganded H122C/H122C ΔCYS PGHS-2 construct, the S121P homodimer construct exhibited distributions of the S1, S2, and S2a in its unliganded state (Fig. 4, *A* and *E*). The presence of the S2a state indicates that the mutation alters the conformation of the 120 to 129 loop prior to ligand binding. Consistent with the functional data, FBP binding failed to shift the cyclooxygenase active site entrances to the tightened state (Fig. 4, *B* and *F*). As expected, AA binding resulted in a distribution of states that mirrored those observed for PA binding to the native homodimer construct (Fig. 4, *C*, *D* and *G*).

**Figure 5:**
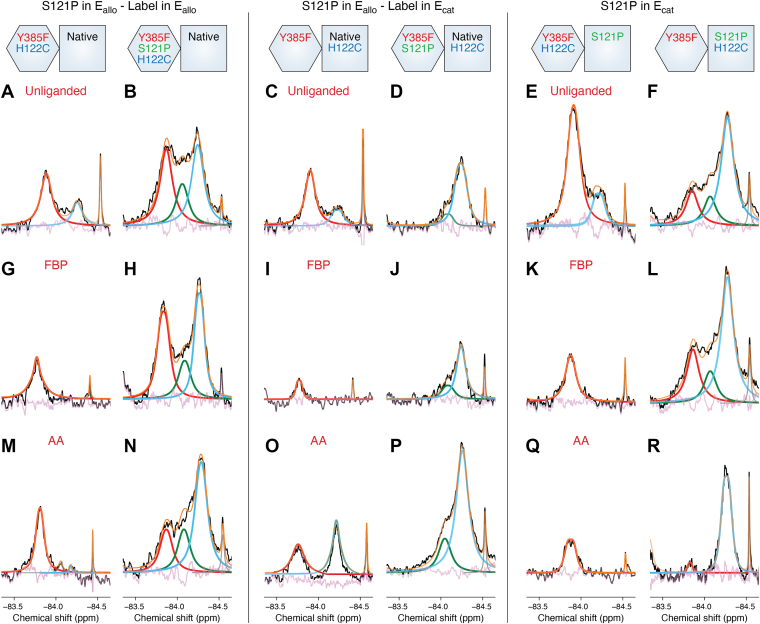
**^19^F-NMR Spectra Derived from S121P Heterodimer Constructs in the Presence of FBP and AA.** The constructs utilized are depicted at the *top* of the figure. Comparison of ensembles arising from native and S121P heterodimer constructs in unliganded (*A*–*F*), FBP-bound (*G*–*L*), and AA-bound (*M-R*) states. Experimental spectra colored, deconvoluted and fit as described in Figure 2.

Heterodimer constructs were subsequently engineered to locate the S121P mutation in either E_allo_ or E_cat_ and subsequently analyzed using ^19^F-NMR. Placement of the S121P mutation in E_allo_ resulted in the observation of S2 and S2a states in E_cat_ of the unliganded enzyme (Fig. 5*D*), further indicating that the conformation of the 120 to 129 loop and cyclooxygenase channel entrance is pre-determined prior to ligand binding. AA binding failed to shift the cyclooxygenase channel entrance of E_allo_ into a tightened state (Fig. 5, *M* and *N*). The binding of FBP to this construct also did not result in a transition to the tightened state (Fig. 5, *H* and *J*), when compared to the native heterodimer construct (Fig. 5, *G* and *I*). In contrast, placement of the S121P mutation in E_cat_ facilitated E_allo_ to undergo the native-like transitions to the tightened state upon binding of FBP and AA (Fig. 5, *G*, *K*, *M* and *Q*). Finally, the predominant S2 states seen in E_cat_ containing the S121P mutation corroborate the findings of attenuated inhibition by FBP and one-third contribution to the increase in V_max_ observed for this heterodimer observed in related functional studies (Fig. 5, *L* and *R*) (17).

## Current model and future directions

**Figure 6:**
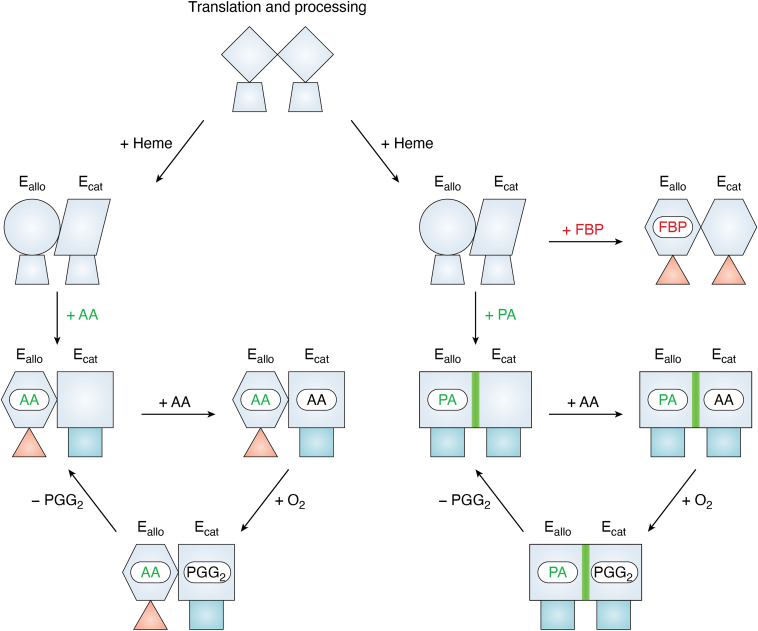
**Conformational States of E_allo_ and E_cat_ and their Roles in the Allosteric Modulation of Cyclooxygenase Activity.** A model linking the ^19^F-NMR derived conformational states of E_allo_ and E_cat_ and the 120 to 129 loop to the allosteric modulation of cyclooxygenase activity by FBP and PA. The unliganded enzyme exists as a conformational heterodimer in solution, with both relaxed and tightened states observed at the cyclooxygenase active site entrance (trapezoid). The binding of AA results in the generation of asymmetry between subunits, with tightened (*red triangle*) and relaxed (*blue rectangle*) states observed at the active site entrances of E_allo_ and E_cat_, respectively. FBP binding to E_allo_ results in the generation of symmetrical tightened states at the active site entrance in both subunits. In contrast, PA binding results in the generation of symmetrical relaxed states at the active site entrances in both subunits, along with an alteration in the conformation of the 120 to 129 loop (*green line*). Adapted with permission from Dong and Malkowski, Biochemistry 64, 1380 to 1392 (2025). Copyright 2025 American Chemical Society.

The experimental data arising from the ^19^F-NMR analyses have revealed how ligand-dependent shifts in conformational ensembles modulate PGHS-2 activity. Elucidation of these conformational ensembles also adds a structural component that nicely complements the previous models derived from functional and biochemical findings (Fig. 6) (26, 30, 31). After translation, processing and the binding of heme, both E_allo_ and E_cat_ fluctuate between tightened and relaxed cyclooxygenase active site entrances in the unliganded enzyme. AA binding imposes asymmetry at the active site entrances – tightened in E_allo_, relaxed in E_cat,_ suggesting that constriction in E_allo_ triggers structural rearrangements across the dimer interface that favor catalysis in E_cat_. The balance is reset by allosteric potentiators and inhibitors: FBP locks both subunits in the tightened state to suppress activity, while PA locks both subunits in the relaxed state to enhance it. From a pure structural perspective, the observed differential states likely reflect differences in how PA and FBP interact with specific regions of the cyclooxygenase active site (19). Utilization of solution-based ^19^F-NMR is the first attempt at bridging the gap between the static information derived from crystal structure analyses and the ensembles governing the regulation of PGHS function. Further characterizations of structural transitions responsible for communication between subunits and validation of the current model will require the application of additional solution-based technologies that probe other regions of the enzyme.

## Dedications

We dedicate this review to the memory of William L. Smith. Bill was trained as a lipid biochemist and spent the majority of his 40+-year career focused on the catalysis and inhibition of PGHS-1 and PGHS-2. The reader is referred to the retrospective that Bill wrote shortly after his retirement to gain insight into the scope and depth of his scientific career (as well as his “dry” sense of humor) (39). Bill started his academic career in the Department of Biochemistry at Michigan State University, where he rose to the rank of full professor and served as department chair. He left Michigan State University in 2003 to become Chair of the Department of Biological Chemistry at the University of Michigan until his retirement in 2015. I was a Postdoctoral Associate in the Smith laboratory at Michigan State from 1997 to 2001 and a collaborator from 2009 to 2015. Liang was a Postdoctoral Fellow in the Smith laboratory from 2009-2015 and subsequently joined my laboratory upon Bill’s retirement. He was an outstanding scientist, as well as a wonderful mentor and colleague to both of us. He will truly be missed.

## Conflict of interest

The authors declare that they have no conflicts of interest with the contents of this article. This article is part of a special issue honoring the memory of William L. Smith.
